# Improvements in Sleep Quality in Patients With Major Depressive and Generalized Anxiety Disorders Treated With Individualized, Parcel‐Guided Transcranial Magnetic Stimulation

**DOI:** 10.1002/brb3.70088

**Published:** 2024-10-17

**Authors:** Si Jie Tang, Jonas Holle, Sirjan Mor, Nicholas B. Dadario, Mark Ryan, Charles Teo, Michael Sughrue, Jacky Yeung

**Affiliations:** ^1^ School of Medicine University of California Davis Medical Center Sacramento California USA; ^2^ Cingulum Health Rosebery Australia; ^3^ Robert Wood Johnson Medical School Rutgers University New Brunswick New Jersey USA; ^4^ Department of Neurosurgery Yale University School of Medicine New Haven Connecticut USA

**Keywords:** personalized, parcel‐guided, repetitive transcranial magnetic stimulation (rTMS), sleep

## Abstract

**Introduction:**

Poor quality sleep has often been cited as a cause of lowered quality of life in patients with affective disorders such as major depressive disorder (MDD) and generalized anxiety disorder (GAD). As sleep and affective disorders are affected by multi‐network interactions, we hypothesize that the modulation of the central executive network (CEN), salience, and default mode networks (DMNs) through individualized repetitive transcranial magnetic stimulation (rTMS) may improve sleep and quality of life.

**Methods:**

A retrospective analysis from 2020 to 2023 was conducted in patients with affective disorders at Cingulum Health. Multiple targets were selected based on anomalies detected from individual, functional connectivity networks from a machine‐learning connectivity software. rTMS was conducted with accelerated continuous or intermittent theta burst stimulation (TBS) based on the anomaly detected. Pittsburgh Sleep Quality Index (PSQI), EuroQol (EQ5D), Beck's Depression Inventory (BDI), and the General Anxiety Disorder‐7 (GAD‐7) questionnaires were administered prior to, after, and at follow‐up of rTMS.

**Results:**

Twenty‐seven patients were identified, and the most common diagnoses were MDD (41%) or MDD with GAD (41%). All patients had at least one rTMS target in the CEN. The most common target (19 patients) was L8Av in the dorsolateral prefrontal cortex (dlPFC). Patients experienced significant improvements in sleep, quality of life, depressive, and anxiety symptoms after rTMS and during follow‐up. Improvements in sleep correlated with quality of life at follow‐up.

**Conclusion:**

This study suggests that personalized, parcel‐guided rTMS is safe and may provide sustained improvements in sleep, quality of life, and affective symptoms for patients with affective disorders.

## Graphical abstract

Our retrospective study found that a personalized parcel‐guided approach to repetitive transcranial magnetic stimulation for the treatment of affective disorders is associated with improvement in sleep quality in addition to affective symptoms and quality of life.

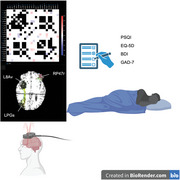

**Abbreviations**
aTBS: accelerated theta burst stimulation
BDI: Beck's Depression Inventory
dlPFC: dorsolateral prefrontal cortex
DMN: default mode network
ECN: central executive network (CEN)/executive control network
EQ5D: EuroQol
GAD: generalized anxiety disorder
GAD‐7: General Anxiety Disorder‐7
MDD: major depressive disorder
OCD: obsessive‐compulsive disorder
PPC: posterior parietal cortex
PSQI: Pittsburgh Sleep Quality Index
PTSD: post‐traumatic stress disorder
rsfMRI: resting‐state functional MRI

## 1 Introduction

Sleep is among the most significant biological processes necessary for maintaining health and a healthy quality of life. It is integral in regulating homeostatic processes and modulating synaptic activity (Lanza, DelRosso, and Ferri 2022). Yet abnormalities in sleep quality are frequently observed in nearly one‐half of all individuals with affective disorders which include, but are not limited to, perturbances in circadian rhythms, wake after sleep onset (WASO) (Chang et al. 2023), lack of REM sleep, and chronic insomnia (Benca et al. 1997). Associations between mood disorders and insomnia have been well studied. These patients have been shown to have disturbances in sleep continuity, shortened REM latency, increased REM sleep duration, and increased REM density (Buysse et al. 2008). There is support for the role sleep plays in the emergence of depressive states (Fang et al. 2019). Despite the well‐characterized link between affective disorders and poor sleep quality, the neural substrates underlying poor sleep quality have been less characterized and difficult to treat.

Recent advancements in the neuroimaging community and high‐throughput approaches have allowed for improved mapping of the human brain connectome—the complete set of connections in the brain—which have provided insight into various neuropsychiatric illnesses and mechanisms of sleep quality. They have revealed several intrinsically linked neural pathways underlying poor sleep quality, affective disorders, and unfavorable health outcomes (Worley 2018). Ultimately, the interaction between three higher order cognitive brain networks, which form an axis within which the other large‐scale brain networks align, is a defining feature in many neuropsychiatric illnesses as well as insleep (Menon 2011). Among these implicated pathways is the default mode network (DMN)—linked to self‐referential information processing—which becomes noticeably disturbed most often in cases of insomnia (Marques et al. 2018). Increased connections within the DMN have been associated with poor sleep quality and sleep deprivation (Tian et al. 2020; Yu et al. 2018). Another notable pathway is the salience network (SN), which plays a critical role in detecting stimuli related to behavior and integrating neural processes (Briggs et al. 2022). Individuals who scored highly on the Pittsburgh Sleep Quality Index (PSQI) score showed a positive correlation between the SN and activity in the left dorsolateral prefrontal cortex (dlPFC) (Cheng et al. 2022). Furthermore, the strength of coupling of the SN and DMN has been implicated in the pressure for sleep during a sleep‐deprived state (Lei et al. 2015). Lastly, the Central Executive Network (CEN) is responsible for a variety of higher cognitive functions ranging from waking memory to decision‐making and problem‐solving. Less variability in resting state functional connectivity between the CEN and SN has been described in patients with insomnia compared to patients without sleep complaints (Wei et al. 2020).

The connections between and within the same networks implicated in sleep—namely, DMN, CEN, and SN—are disrupted in affective disorders. It is believed that disruption of these “cognitive control networks” leads to an inappropriate allocation of cognitive resources between active (CEN) and passive (DMN) states of mind (Menon 2011). Altered connectivity between these networks has been implicated in patients with generalized anxiety disorder (GAD), major depressive disorder (MDD), obsessive‐compulsive disorder (OCD), and many more neuropsychiatric illnesses (Tian et al. 2020; Xiong, Guo, and Shi 2020; Fan et al. 2017). Given the tight and often bidirectional relationship between mood, anxiety, and sleep, it is possible that many of these connectomic circuits and biological mechanisms also underlie poor sleep quality and therefore can be similarly targeted. In affective disorders, modulatory treatments targeting these underlying networks based on individual connectivity abnormalities have shown significant promise in the ability to normalize these network disruptions and therefore result in improved mood (Young et al. 2023). Thus, the ability to noninvasively alter these networks has also provided a significant opportunity to attempt to treat poor sleep quality directly in patients with affective disorders as well.

Transcranial magnetic stimulation (TMS) is a well‐established and safe noninvasive brain stimulation technique (Rossi et al. 2009). TMS applies a magnetic pulse extracranially to generate an electrical current in the cortex and produce electrophysiological changes in a specific target area and connected brain regions. It is an FDA‐approved treatment for many neuropsychiatric illnesses, including MDD and OCD. Recently, TMS has grown as an attractive treatment for sleep disorders, such as insomnia, obstructive sleep apnea, restless leg syndrome, and sleep deprivation (Lanza et al. 2023). TMS has been used for poor sleep quality due to hypotheses concerning similar underlying neuroanatomic substrates. TMS treatment in these patients has been shown to normalize connectivity disturbances between the DMN and the insula resulting in improved sleep quality (Cheng et al. 2022). However, the effects of repetitive transcranial magnetic stimulation (rTMS) on sleep quality in patients with affective disorders remain unclear, with some studies reporting robust improvements on standardized sleep scales compared to sham treatment, whereas other controlled studies report no improvement in sleep quality but only mood (Collins et al. 2022; Rosenquist et al. 2013; Antczak et al. 2017). Reasons for these mixed results are likely in part due to using traditional craniometric methods of targeting the dlPFC which poorly treats the exact connections that may be disrupted in an individual and vary according to craniometric coordinates. By treating all individuals the same with this method, the same cranial location may be targeted but in fact modulate very different underlying connections leading to poor treatment response, as has been shown elsewhere (Rosen et al. 2021). Thus, a more specific and individualized targeting method for TMS treatment is necessary to truly treat poor sleep quality on an individual basis.

Up until recently, a tool to visualize and quantify a person's functional connectomics was not readily available. A personalized, parcel‐guided approach to rTMS allows us to target multiple neuropsychiatric symptoms based on a network model of disorder psychophysiology and in a more anatomically specific manner between patients (Moreno‐Ortega et al. 2020). Using the same approach, we have demonstrated promising preliminary results in treating various neuropsychiatric disorders, including anxiety, ischemic stroke, and neurorehabilitation after brain tumor surgery (Young et al. 2023; Chen et al. 2023; Tang et al. 2022; Yeung et al. 2021). This approach allows clinicians to treat symptoms and specific circuits of an individual patient rather than using a cookie‐cutter intervention for treatment of affective disorders (Tang et al. 2022). As the same networks implicated in a range of affective disorders are also related to insomnia, we hypothesized a priori that rTMS used for treating affective disorders may be associated with improvement in sleep quality, neuropsychiatric symptoms, and quality of life.

Here, we present a case series of personalized rTMS treatment for 27 patients with affective disorders and observe their sleep quality metrics collected in a retrospective manner. We observed that our protocol for the treatment of affective disorders was associated with improved quality of life and sleep within the first week with sustained effects during the follow‐up visits. Unlike previous work, which has found variable results after rTMS for sleep quality, this study utilized a novel parcel‐guided rTMS treatment based on individualized connectivity analysis. Through unbiased connectivity analysis to identify dysfunctional regions, patient‐specific targets were identified that varied between patients and, following stimulation, resulted in significant improvements in sleep quality and quality of life metrics.

## 2 Methods

### 2.1 Subjects

A retrospective analysis was performed of patients who were treated for affective disorders at Cingulum Health (Sydney, Australia) from 2020 to 2023, inclusive. Patients were included in the study if they were diagnosed with an affective disorder, which included MDD, generalized anxiety disorder (GAD), post‐traumatic stress disorder (PTSD), or OCD based on the Diagnostic and Statistical Manual of Mental Disorders‐V (DSM‐V).

Patients were included if a qualified psychiatrist or primary care physician diagnosed them with an affective disorder according to DSM‐V criteria and if they were at least 18 years of age.

Patients were excluded if they were not able to undergo functional imaging due to claustrophobia or unable to participate in rTMS due to contraindications such as metal in the brain, surgically implanted neurostimulators, or high risk of seizures (epilepsy). Patients who completed TMS were excluded from retrospective analysis if they did not return for a follow‐up consultation.

The nature and potential risks and side effects of rTMS were explained to the patient prior to being given informed consent. Off‐label usage of rTMS for conditions other than MDD and OCD was thoroughly discussed with the patients during the consent process. All patients were carefully screened for any contraindications, such as a seizure risk, for being a candidate for rTMS.

### 2.2 Affective Disorders and Quality of Life Questionnaires

All patients were administered the EuroQol (EQ5D‐5L) and PSQI prior to treatment, after treatment, and follow‐up. Follow‐up consultation occurred during a minimum interval of at least 1 month after treatment. Patients diagnosed with MDD or GAD were administered the Beck's Depression Inventory (BDI; version BDI‐II) and the General Anxiety Disorder‐7 (GAD‐7) questionnaire, respectively. Patients who had a BDI lower than 12 were considered to be in remission (Riedel et al. 2010). The minimal clinically important difference (MCID) in GAD‐7 is four points (Toussaint et al. 2020). For patients who were diagnosed with PTSD, the PTSD checklist (PCL‐5) was administered during those same time periods. For patients who were diagnosed with OCD, the Yale Brown Obsessive‐Compulsive Scale (YBOCS) was administered at the same time periods.

### 2.3 Personalized Brain Atlas

**FIGURE 1:**
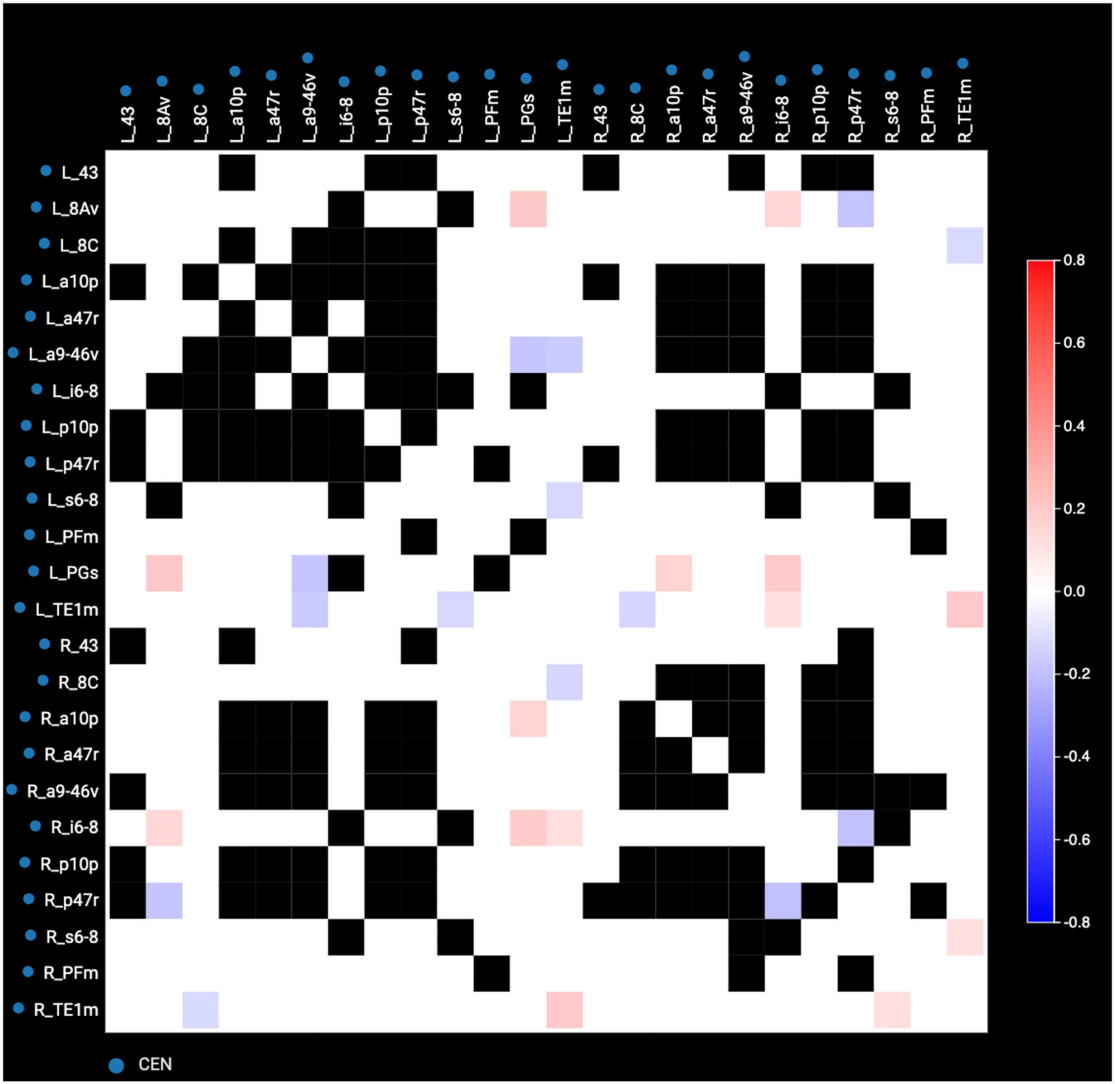
Anomaly matrix of the CEN of Patient 1. Anomaly matrices display connectivity between sets of parcellations. White squares indicate connectivity within normal range, and black squares represent connectivity that is too variable to determine a normal range. Red represents hyperconnectivity, and blue represents hypoconnectivity. The degree of connectedness is indicated by the hue of the red or blue as described in the key on the right‐hand side of the matrix.

Resting‐state functional MRI (rsfMRI) and non‐contrast T1‐weighted images were collected as described previously on a Philips 3T Achieva (Tang et al. 2022). Resting state preprocessing analysis was performed as previously described (Yeung et al. 2021). Correlation and anomaly detection with the Omniscient Infinitome software (Sydney, Australia) were performed to create anomaly detection matrices for each patient (Figure 1) and compared against a normative atlas consisting of rsfMRI data of 200 healthy controls from the OpenNeuro (https://openneuro.org/) and SchizConnect (http://schizconnect.org) datasets. Connectivity anomalies greater than three sigmas from the normal population were detected, and a target selection was performed with the initial hypothesis that functional connectivity anomalies between the DMN, salience, and CEN were drivers for affective disorders. Importantly, the selection of targets was on the basis of the detection of connectivity anomalies, and no conscious, subjective guidance was provided in the determination of the anomaly matrices. The results from the anomaly detection algorithm were then used to guide the selection of three target regions and iTBS or cTBS was prescribed based on hypoconnectivity and hyperconnectivity, respectively, compared to the 200 normal subjects (Huang et al. 2005; Jung and Lambon Ralph 2021).

### 2.4 rTMS Treatment

All rTMS was conducted based on an accelerated theta burst stimulation (aTBS) protocol with a Magventure MagPro X100 TMS machine with a butterfly cool coil (Alpharetta, USA). Targets at cortical depths greater than 30 mm were assumed to be out of range of the effective field depth and focality of the particular coil used (Deng, Lisanby, and Peterchev 2013).

The position of the coil and the patient's head was tracked using Localite stereotactic neuronavigation system (Bonn, Germany). This system provided real‐time feedback as to the location of the stimulation device in relation to the patient's selected targets superimposed on T1 images of the cortex.

Five image‐guided theta burst stimulation (TBS) treatment sessions per day for 5 days with 1‐h time gaps between sessions were conducted. iTBS was performed at bursts of 3‐pulse 50‐Hz bursts given every 200 ms at (5 Hz) for 40 trains, with an inter‐train interval of 6.3 s, for a total of 1200 pulses. cTBS was performed as one train of 600 stimuli applied in 3‐pulse 50‐Hz bursts given every 200 ms (5 Hz), for a total of 1800 pulses (Tang et al. 2022). Targets were stimulated at 80% of resting motor threshold.

### 2.5 Statistical Analysis

Mixed‐effects analyses with Dunnett's muliple comparisons test and correlation analyses were performed using GraphPad Prism 9. Significance was defined as a *p* value < 0.05.

## 3 Results

Twenty‐seven patients met criteria for our study (Table 1). The average age of patients was (mean ± standard deviation) 44 ± 16 years. Patients had an average duration of 13 ± 11 years of symptoms from diagnosis of their affected disorder to rTMS treatment. The average follow‐up time was 68.5 ± 69.5 days. Seven patients did not complete follow‐up assessments during their follow‐up consultation and thus were not included in the follow‐up analysis but were still included in the posttreatment analysis.

**TABLE 1:** Patient characteristics.

Characteristic		Patients (*N* = 27) (%)
**Diagnosis**		
MDD		11 (41)
MDD and GAD		11 (41)
MDD and ADHD		1 (4)
GAD		2 (7)
GAD and OCD		1 (4)
GAD and PTSD		1 (4)
**Demographics**		(Mean ± SD) [range]
Age (years ± SD)		44 ± 16
Duration of affective disorder (years)		13 ± 11
PSQI baseline score (Scale 0–21)		9 ± 3 [3–16]
EQ‐5D baseline score (Scale −0.594–1)		0.503 ± 0.243 [−0.012 to 0.879]
BDI baseline score (Scale 0–63)		25.5 ± 8.6 [11–43]
GAD‐7 baseline score (Scale 0–21)		13.4 ± 4.7 [7–21]
Sex	Male—no. (%) Female—no. (%)	17 (63) 10 (37)

Abbreviations: GAD, generalized anxiety disorder; MDD, major depressive disorder; OCD, obsessive‐compulsive disorder; PSQI, Pittsburgh Sleep Quality Index; PTSD, post‐traumatic stress disorder.

### 3.1 Common Targets of rTMS

**FIGURE 2:**
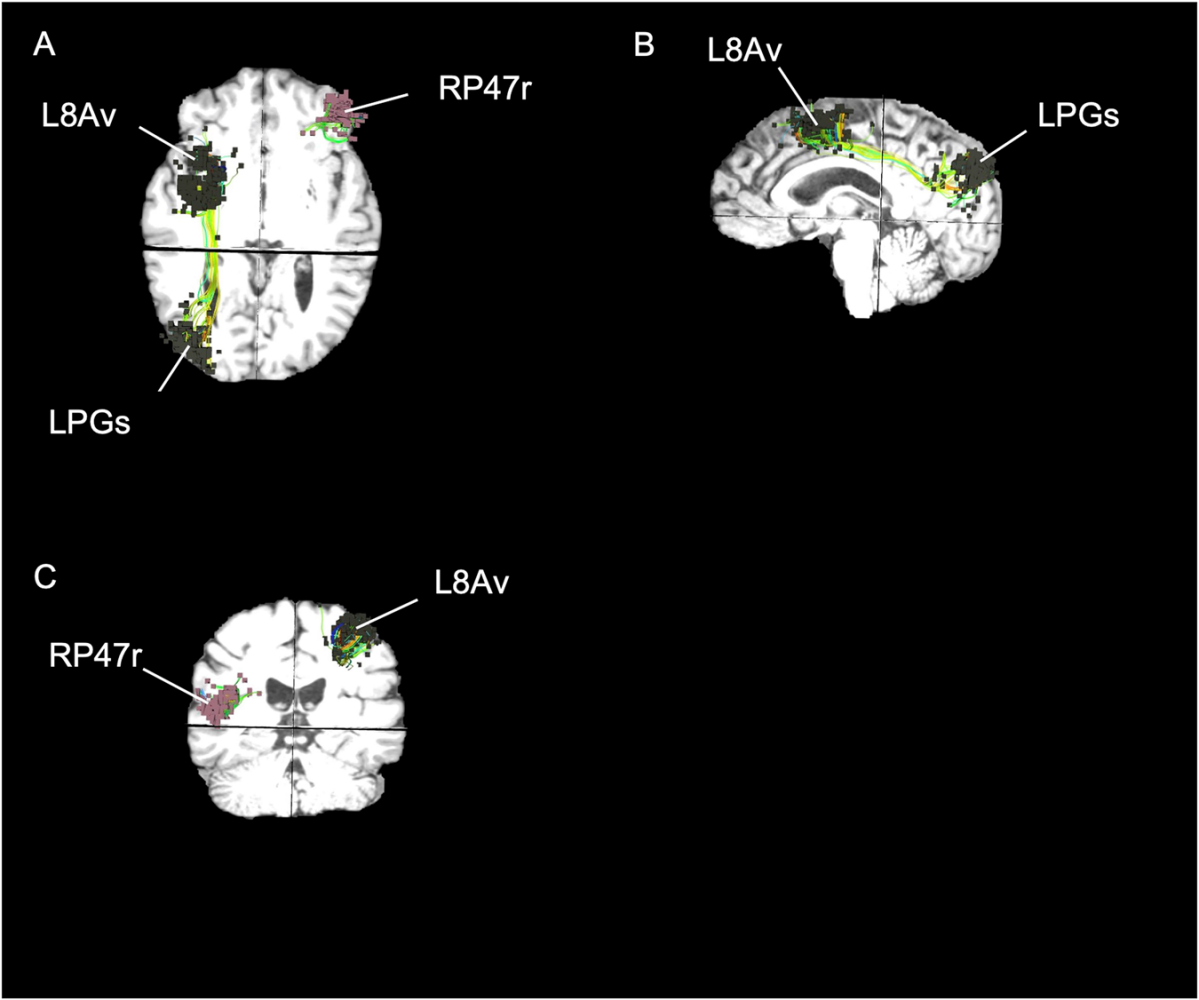
Anatomical locations of rTMS for Patient 1. (A) Axial, (B) sagittal, and (C) coronial visualization of rTMS targets superimposed on top of MRI of Patient 1. Patient 1 is a 48‐year‐old female with a diagnosis of MDD for 10 years. She had a baseline score of 8, posttreatment of 9, and follow‐up of 3 on the PSQI.

Table 2 details the common targets in this cohort of patients. Location targets are described based on the Glasser Atlas (Glasser et al. 2016). Clinical details for each patient and their rTMS targets can be found in Figure S1. The targeted regions for Patient 1 are shown in Figure 2 as an example of rTMS targets. For all patients except 2, 11, and 14, all rTMS targets were part of the CEN. Those three patients also had at least one target in the CEN, but other networks such as the salience, visual, and language networks were included. No targets in the DMN were observed in this cohort. Nineteen patients had a target at the L8Av, which was the most common target shared by this cohort of patients. This area is within the dlPFC and is within the CEN. The second most common was LPGs, which was shared by 14 patients. LPGs are located in the inferior parietal cortex and are also within the CEN.

**TABLE 2:** Patient percentage with each target region.

Location (sequence)	Full name	Location	Network	Number of patients with this target (%) Total targets = 79
L46	Left area 46	Brodmann area 46	Salience	1 (1)
L43	Left area 43	Brodmann area 43	CEN	1 (1)
L6ma	Left area 6m anterior	Supplementary motor area	Sensorimotor	1 (1)
L8Av	Left area 8Av	Dorsolateral prefrontal cortex	CEN	19 (24)
LPFm	Left area PFm complex	Inferior parietal cortex	CEN	9 (11)
LPGs	Left area PGs	Inferior parietal cortex	CEN	14 (18)
LPHT	Left parahippocampal temporal	Lateral temporal cortex	Language	1 (1)
Ls6‐8	Left superior 6–8 transition Area	Dorsolateral prefrontal cortex	CEN	7 (9)
LTe1m	Left area TE1 middle	Lateral temporal cortex	CEN	9 (11)
LV4	Left visual area 4	Visual cortex	Visual	1 (1)
Rp47r (iTBS)	Right area posterior 47r	Dorsolateral prefrontal cortex	CEN	1 (1)
RPFm	Right area PFm complex	Inferior parietal cortex	CEN	2 (3)
Rs6‐8	Right superior 6–8 transition area	Dorsolateral prefrontal cortex	CEN	3 (4)
RTe1m	Right area TE1 middle	Lateral temporal cortex	CEN	10 (13)

*Note*: The number of patients with each target parcellation. Target locations based on parcellations described in Glasser's Atlas.

Abbreviation: CEN, central executive network.

### 3.2 Changes in Sleep Quality as Related to Quality of Life, Depression, and Anxiety

**FIGURE 3:**
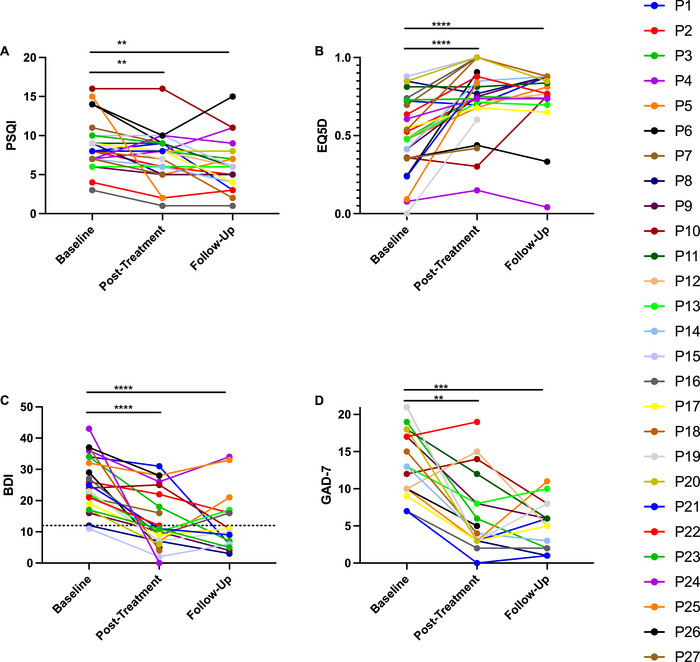
Questionnaires on quality of life, sleep, and affective disorders. PSQI (Scale 0–21), EQ5D (Scale −0.594–1), BDI (Scale 0–63), and GAD‐7 (Scale 0–21) scores at baseline, after treatment, and during 1‐month follow‐up. (A) In the PSQI, there was improvement of sleep based on the PSQI after treatment (*p *= 0.0225, mixed‐effects analysis, Dunnett's multiple comparisons test, *N* = 27) and during follow‐up (*p *= 0.0005, mixed‐effects analysis, Dunnett's multiple comparisons test, *N *= 20) compared to baseline. (B) In the EQ5D, there was improvement after treatment (*p *< 0.0001, mixed‐effects analysis, Dunnett's multiple comparisons test, *N* = 27) and during follow‐up (*p *< 0.0001, mixed‐effects analysis, Dunnett's multiple comparisons test, *N* = 20) compared to baseline. (C) In the BDI, there was improvement after treatment (*p *< 0.0001, mixed‐effects analysis, Dunnett's multiple comparisons test, *N* = 23) and during follow‐up (*p *< 0.0001, mixed‐effects analysis, Dunnett's multiple comparisons test, *N* = 16) compared to baseline. Dotted line corresponds to the BDI remission score. (D) In the GAD‐7, there was improvement after treatment (*p *= 0.0002, mixed‐effects analysis, Dunnett's multiple comparisons test, *N* = 19) and during follow‐up (*p *< 0.0001, mixed‐effects analysis, Dunnett's multiple comparisons test, *N* = 15) compared to baseline. BDI, Beck's Depression Inventory; EQ5D, EuroQol; GAD‐7, General Anxiety Disorder‐7; PSQI, Pittsburgh Sleep Quality Index.

Patients reported improvements in quality of life, depression, and anxiety after rTMS treatment and during follow‐up compared to baseline (Figure 3A–D). As a group, there was an improvement of sleep on the basis of the PSQI after treatment and during follow‐up compared to baseline (Figure 3A). There was an improvement of quality of life (QoL) on the basis of EQ5D score after treatment and during follow‐up (Figure 3B). BDI scores were improved after treatment and during follow‐up compared to baseline (Figure 3C). There was an improvement in GAD‐7 after treatment and during follow‐up compared to baseline (Figure 3D).

The correlation between PSQI scores and EQ‐5D, BDI, and GAD‐7 scores is presented in Figure S2A–I. At baseline and posttreatment, PSQI was not significantly correlated with EQ‐5D, BDI, or GAD‐7. At follow‐up, PSQI was significantly correlated with EQ‐5D and BDI but not GAD‐7.

For Patient 12 who was additionally diagnosed with PTSD and Patient 11 with OCD, PCL5 and YBOCS were administered, respectively, for each patient. From a baseline of 45 on the PCL5, Patient 12 reported improvements in PTSD symptoms with a posttreatment score of 49 and a 1‐month follow‐up improvement of 53% to a score of 21. Patient 11 did not report improvements in his OCD; his YBOCS remained at 20 at posttreatment and follow‐up from a baseline of 21.

### 3.3 Safety

Patients reported minor side effects, including fatigue, headaches, muscular twitching, and discomfort. The largest number of patients reported fatigue (12/27, 44%) and muscular twitching (10/27, 37%). Five individuals reported headaches (19%), and four individuals reported discomfort (15%). Five individuals did not report any symptoms at all.

## 4 Discussion

This study in 27 patients has demonstrated that parcel‐guided, rTMS treatment may improve sleep quality, QoL, and symptoms of affective disorders. The treatment modality described here uses an unbiased approach to rTMS target selections based on individualized connectivity analyses.

CEN was the most common target network identified in this retrospective study. This network includes the posterior parietal cortex (PPC) and dlPFC which allocate cognitive resources to changing environmental demands. An important feature of MDD is the disruption of goal‐directed cognitive tasks that include working memory, cognitive flexibility, emotional processing, and emotional regulation, all of which are critical functions of the CEN (Elderkinthompson et al. 2007, Joormann and Gotlib 2008). Therefore, it is clear that network integrity of the CEN is highly essential in contributing to the improvement of MDD. Literature has also shown that MDD patients have enhanced influences within the CEN but attenuated influences from CEN to DMN (Tian et al. 2020). Similarly, in our study, we have consistently found anomalies in the CEN region of our MDD patients compared to controls and chosen to target those regions of hyperconnectivity with cTBS.

The use of rTMS, and primarily cTBS in our study, led to improvements in sleep and certain affective symptoms. Whether rTMS is improving affective symptoms leading to better sleep or better sleep is decreasing affective symptoms is difficult to conclude from this study.

Previously, rTMS and its sleep effects have been heavily studied in chronic primary insomnia. When compared to patients treated with medication and psychotherapy, patients treated with low‐frequency rTMS in the dlPFC and PPC had the lowest relapse and recurrence rates within 3 months. Specifically, rTMS improved Stage III sleep and REM sleep cycle compared with the control groups in primary insomnia (Sonmez et al. 2020). rTMS has also shown improvement in patients with restless leg syndrome, obstructive sleep apnea, narcolepsy, hypersomnia, and sleep bruxism (Sonmez et al. 2019; Nardone et al. 2020; Zhou et al. 2016). Moreover, some sleep disorders, such as periodic leg movements during sleep (PLM), have been found to be associated with antidepressants, making rTMS an attractive alternative to antidepressants with such sleep disorders (Ferri et al. 2023).

rTMS is a continually evolving therapy, and the inclusion of TBS has shown advantages over conventional rTMS in the treatment of MDD (Voigt, Leuchter, and Carpenter 2021). However, little is known about the sleep benefits in these patients. A pilot study demonstrated that rTMS was found to decrease hypersomnia in adolescents with treatment‐resistant MDD but did not improve insomnia (Sonmez et al. 2020). In our patients, however, sleep significantly improved after treatment and during follow‐up compared to baseline using the PSQI scale.

Moreover, our patients found significant improvement in their depression symptoms after a week of therapy and during their follow‐ups. Depression and anxiety often coexist, and the lifetime comorbidity rate of the two syndromes has been reported at 90% (Gorman 1996; Chen et al. 2019). Treating symptoms of both disorders at once is an important aspect of our protocol, and witnessing simultaneous significant improvement in sleep quality invites investigation into the relationship between rTMS, sleep disturbance, and the symptoms of affective disorder. Although our preliminary results are not enough to suggest that rTMS can be used to treat sleep disorders, they suggest that further investigation into the use of personalized rTMS for sleep problems secondary to affective disorders is required. Ultimately, future studies are needed to fully elucidate sleep benefits of rTMS in patients with affective disorders.

### 4.1 Personalized Approach in Identifying rTMS Targets

Individuals and their symptoms are often classified into an affective disorder or primary insomnia disorder based on the DSM. Many patients experience a range of symptoms: insomnia, hypersomnia, lack of appetite, hyperphagia, anhedonia, sadness, anxiety, psychomotor agitation, and psychomotor retardation to suicidal or homicidal ideation. These symptoms have their primary basis in connectivity dysfunction on an individual level (Siddiqi et al. 2020). For primary insomnia disorder, increased functional connectivity has been observed within the DMN, SN, sensory‐motor network, and dorsal‐attentional network (Fasiello et al. 2022). One study found increased SN activation in patients with MDD and insomnia (Qi et al. 2022). The SN is known to modulate the activation and deactivation of the DMN and CEN. Thus, dysfunction in the SN may lend a decreased response to changing environments in people with insomnia disorder (Wei et al. 2020).

Decreased functional connectivity in frontoparietal network, also known as the CEN, is seen in depression, anxiety, and post‐insomnia negative emotions (Fasiello et al. 2022). This suggests the role of CEN in insomnia disorder etiology. Primary insomnia patients have abnormalities in the right frontoparietal network (CEN) and right parietal lobe (Li et al. 2018; Zhou et al. 2017). One study specifically targeted the right parietal cortex for the treatment of primary insomnia with low‐frequency rTMS. The findings were significant for improvement in insomnia symptoms, normalization of time‐varying EEG signals in patients, and curative effect that can last for 1 month (Song et al. 2019). With evidence emerging toward connectivity dysfunction in insomnia, it is imperative to better characterize and treat specific patient needs with an individualized approach such as rTMS.

Given the shared network alterations of the DMN, CEN, and SN in sleep and neuropsychiatric disorders, it makes sense to utilize a connectomics approach to develop a targeted treatment for individuals with sleep and mental health complaints. Our study further highlights that a transdiagnostic method to clinical intervention will allow precise treatment of insomnia by targeting underlying network dysfunctions. The two most frequent targets, L8Av and LPGs, are part of the CEN and are located in the dlPFC and the inferior parietal cortex, respectively. For 24 of 27 patients, all targets were in the CEN. Only one patient had a target in the SN, which was parcellation L46.

Our personalized brain mapping approach allows the measurement of abnormalities in functional connectivity on an individual level. It has previously been published as a promising approach to rehabilitation in patients following craniotomy and stroke (Tang et al. 2022; Yeung et al. 2021). With this technology, an a priori hypothesis—namely, that network alterations in these three pathways are implicated in sleep and mood disorders—can then be utilized to identify individual hypo‐ and hyperconnectivities compared to healthy controls. Parcellations with abnormal connectivity to other parcellations are then identified on the anomaly matrix, and such areas are then targeted by rTMS treatment. This approach accounts for individualized connectivity differences among each patient and facilitates a multiple brain region targeting approach for treatments of affective disorders where the disorder is not implicated in one specific region of the brain.

### 4.2 Safety of Personalized rTMS

No adverse side effects, such as seizures, were experienced by patients following rTMS treatment. The most common side effects were fatigue and muscle twitching, both of which are common and have been previously reported in rTMS therapy (Tang et al. 2022; O'reardon et al. 2007).

### 4.3 Limitations

Our study is limited by a lack of randomization as well as the length of time for follow‐up. It is certainly possible that the remission rate we observe is due to the placebo effect as there is a 37%–39% response rate observed for the placebo effect in sham‐controlled rTMS for patients with MDD, and a 74% effect size of active rTMS was produced by sham rTMS for the treatment of insomnia (Jiang et al. 2019; Li et al. 2019; Yesavage et al. 2018). If indeed the results were a placebo effect, the self‐reported scores would have diminished or gone back to baseline during follow‐up because affective symptoms such as depression exhibit placebo response earlier and then gradually reduce with time (Li et al. 2019). However, it is worth mentioning that patients reported continued improvements in quality of life during their 1‐month follow‐up, which is inconsistent with the trajectory of a placebo effect. Nevertheless, longer follow‐up times are warranted to address the effect of placebo. A double‐blinded, sham‐controlled trial is therefore the next step to determine the efficacy of parcel‐guided rTMS for the treatment of affective disorders.

Moreover, as this is a retrospective study, the patient population was limited by the types of patients seen in the clinic. Many of the patients had either MDD and/or GAD. Further studies should be done to see if improvements in sleep are observed in other affective disorders.

## 5 Conclusion

The personalized, parcel‐guided rTMS approach for the treatment of affective disorders may be associated with improvements in sleep quality outcomes. These results invite further investigation with a placebo group to determine the efficacy of rTMS for the improving sleep quality secondary to affective disorders and exploring the additional effects of a personalized multimodal sleep intervention.

## Author Contributions

**Si Jie Tang**: investigation, writing–original draft, visualization, writing–review and editing, formal analysis. **Jonas Holle**: data curation, writing–original draft, investigation, visualization, writing–review and editing. **Sirjan Mor**: writing–review and editing. **Nicholas B. Dadario**: writing–review and editing. **Mark Ryan**: data curation, writing–review and editing, investigation. **Charles Teo**: supervision, methodology. **Michael Sughrue**: supervision, methodology. **Jacky Yeung**: supervision, conceptualization, writing–review and editing, writing–original draft, investigation.

## Ethics Statement

The study was approved by the Human Research Ethics Committee of the South Eastern Sydney Local Health District (2022/ETH00139).

## Conflicts of Interest

Charles Teo and Michael Sughrue are founders of Omniscient Neurotechnology and Cingulum Health. Jonas Holle is an employee of Cingulum Health. Mark Ryan is a contractor at Cingulum Health. Si Jie Tang, Jacky Yeung, Sirjan Mor, and Nicholas B. Dadario do not report any conflicts of interest.

### Peer Review

The peer review history for this article is available at https://publons.com/publon/10.1002/brb3.70088.

## Declarations of Clinical Trial

This manuscript is not a clinical trial.

## Supporting information



Figure S1 Clinical details and rTMS targets.
Figure S2 Correlation of PSQI with EQ‐5D, BDI, and GAD‐7.

## Data Availability

The data that support the findings of this study are available on request from the corresponding author. The data are not publicly available due to privacy or ethical restrictions.

## References

[brb370088-bib-0001] Antczak, J. , A. Poleszczyk , A. Wichniak , M. Rakowicz , and T. Parnowski . 2017. “The Influence of the Repetitive Transcranial Magnetic Stimulation on Sleep Quality in Depression.” Psychiatria Polska 51, no. 5: 845–857. 10.12740/PP/68503. 29289965

[brb370088-bib-0002] Benca, R. M. , M. Okawa , M. Uchiyama , et al. 1997. “Sleep and Mood Disorders.” Sleep Medicine Reviews 1, no. 1: 45–56. 10.1016/s1087-0792(97)90005-8. 15310523

[brb370088-bib-0003] Briggs, R. G. , I. M. Young , N. B. Dadario , et al. 2022. “Parcellation‐Based Tractographic Modeling of the Salience Network Through Meta‐Analysis.” Brain and Behavior 12, no. 7: e2646. 10.1002/brb3.2646. 35733239 PMC9304834

[brb370088-bib-0004] Buysse, D. J. , J. Angst , A. Gamma , V. Ajdacic , D. Eich , and W. Rössler . 2008. “Prevalence, Course, and Comorbidity of Insomnia and Depression in Young Adults.” Sleep 31, no. 4: 473–480. 10.1093/sleep/31.4.473. 18457234 PMC2279748

[brb370088-bib-0005] Chang, F. , E. C. Berenz , O. Ajilore , et al. 2023. “Actigraphic Wake After Sleep Onset and Symptom Severity Correspond With Rumination in Trauma‐Exposed Individuals.” Brain Sciences 13, no. 1: 139. 10.3390/brainsci13010139. 36672120 PMC9856627

[brb370088-bib-0006] Chen, L. , A.‐R. Hudaib , K. E. Hoy , and P. B. Fitzgerald . 2019. “Is rTMS Effective for Anxiety Symptoms in Major Depressive Disorder? An Efficacy Analysis Comparing Left‐Sided High‐Frequency, Right‐Sided Low‐Frequency, and Sequential Bilateral rTMS Protocols.” Depression and Anxiety 36, no. 8: 723–731. 10.1002/da.22894. 30958907

[brb370088-bib-0007] Chen, R. , N. B. Dadario , B. Cook , et al. 2023. “Connectomic Insight Into Unique Stroke Patient Recovery After rTMS Treatment.” Frontiers in Neurology 14: 1063408. 10.3389/fneur.2023.1063408. 37483442 PMC10359072

[brb370088-bib-0008] Cheng, Y. , T. Xue , F. Dong , et al. 2022. “Abnormal Functional Connectivity of the Salience Network in Insomnia.” Brain Imaging Behavior 16, no. 2: 930–938. 10.1007/s11682-021-00567-9. 34686967

[brb370088-bib-0009] Collins, A. R. , J. Cheung , P. E. Croarkin , B. P. Kolla , and S. Kung . 2022. “Effects of Transcranial Magnetic Stimulation on Sleep Quality and Mood in Patients With Major Depressive Disorder.” Journal of Clinical Sleep Medicine 18, no. 5: 1297–1305. 34931606 10.5664/jcsm.9846 PMC9059593

[brb370088-bib-0010] Deng, Z. D. , S. H. Lisanby , and A. V. Peterchev . 2013. “Electric Field Depth‐Focality Tradeoff in Transcranial Magnetic Stimulation: Simulation Comparison of 50 Coil Designs.” Brain Stimulation 6, no. 1: 1–13. 10.1016/j.brs.2012.02.005. 22483681 PMC3568257

[brb370088-bib-0011] Elderkinthompson, V. , J. Mintz , E. Haroon , H. Lavretsky , and A. Kumar . 2007. “Executive Dysfunction and Memory in Older Patients With Major and Minor Depression.” Archives of Clinical Neuropsychology 22, no. 2: 261–270. 10.1016/j.acn.2007.01.021. 17443924

[brb370088-bib-0012] Fan, J. , M. Zhong , J. Gan , et al. 2017. “Altered Connectivity Within and Between the Default Mode, Central Executive, and Salience Networks in Obsessive‐Compulsive Disorder.” Journal of Affective Disorders 223: 106–114. 10.1016/j.jad.2017.07.041. 28743059

[brb370088-bib-0013] Fang, H. , S. Tu , J. Sheng , and A. Shao . 2019. “Depression in Sleep Disturbance: A Review on a Bidirectional Relationship, Mechanisms and Treatment.” Journal of Cellular and Molecular Medicine 23, no. 4: 2324–2332. 10.1111/jcmm.14170. 30734486 PMC6433686

[brb370088-bib-0014] Fasiello, E. , M. Gorgoni , S. Scarpelli , V. Alfonsi , L. Ferini Strambi , and L. de Gennaro . 2022. “Functional Connectivity Changes in Insomnia Disorder: A Systematic Review.” Sleep Medicine Reviews 61: 101569. 10.1016/j.smrv.2021.101569. 34902821

[brb370088-bib-0015] Ferri, R. , M. P. Mogavero , O. Bruni , D. L. Picchietti , and L. M. Delrosso . 2023. “Periodic Leg Movements During Sleep Associated With Antidepressants: A Meta‐Analysis.” Neuroscience and Biobehavioral Reviews 148: 105126. 10.1016/j.neubiorev.2023.105126. 36914081

[brb370088-bib-0016] Glasser, M. F. , T. S. Coalson , E. C. Robinson , et al. 2016. “A Multi‐Modal Parcellation of Human Cerebral Cortex.” Nature 536, no. 7615: 171–178. 10.1038/nature18933. 27437579 PMC4990127

[brb370088-bib-0017] Gorman, J. M. 1996. “Comorbid Depression and Anxiety Spectrum Disorders.” Depression and Anxiety 4, no. 4: 160–168. 10.1002/(SICI)1520-6394(1996)4:4<160::AID-DA2>3.0.CO;2-J. 9166648

[brb370088-bib-0018] Huang, Y.‐Z. , M. J. Edwards , E. Rounis , K. P. Bhatia , and J. C. Rothwell . 2005. “Theta Burst Stimulation of the Human Motor Cortex.” Neuron 45, no. 2: 201–206. 10.1016/j.neuron.2004.12.033. 15664172

[brb370088-bib-0019] Jiang, B. , D. He , Z. Guo , Q. Mu , and L. Zhang . 2019. “Efficacy and Placebo Response of Repetitive Transcranial Magnetic Stimulation for Primary Insomnia.” Sleep Medicine 63: 9–13. 10.1016/j.sleep.2019.05.008. 31600660

[brb370088-bib-0020] Joormann, J. , and I. H. Gotlib . 2008. “Updating the Contents of Working Memory in Depression: Interference From Irrelevant Negative Material.” Journal of Abnormal Psychology 117, no. 1: 182–192. 10.1037/0021-843X.117.1.182. 18266496

[brb370088-bib-0021] Jung, J. , and M. A. Lambon Ralph . 2021. “The Immediate Impact of Transcranial Magnetic Stimulation on Brain Structure: Short‐Term Neuroplasticity Following One Session of cTBS.” Neuroimage 240: 118375. 10.1016/j.neuroimage.2021.118375. 34245868 PMC8456691

[brb370088-bib-0022] Lanza, G. , L. M. DelRosso , and R. Ferri . 2022. “Sleep and Homeostatic Control of Plasticity.” Handbook of Clinical Neurology 184: 53–72. 10.1016/B978-0-12-819410-2.00004-7. 35034758

[brb370088-bib-0023] Lanza, G. , F. Fisicaro , M. Cantone , et al. 2023. “Repetitive Transcranial Magnetic Stimulation in Primary Sleep Disorders.” Sleep Medicine Reviews 67: 101735. 10.1016/j.smrv.2022.101735. 36563570

[brb370088-bib-0024] Lei, Y. , Y. Shao , L. Wang , et al. 2015. “Large‐Scale Brain Network Coupling Predicts Total Sleep Deprivation Effects on Cognitive Capacity.” PLoS ONE 10, no. 7: e0133959. 10.1371/journal.pone.0133959. 26218521 PMC4517902

[brb370088-bib-0025] Li, F. , M. Nasir , B. Olten , and M. H. Bloch . 2019. “Meta‐Analysis of Placebo Response in Adult Antidepressant Trials.” CNS Drugs 33, no. 10: 971–980. 10.1007/s40263-019-00662-y. 31573058

[brb370088-bib-0026] Li, S. , J. Tian , M. Li , et al. 2018. “Altered Resting State Connectivity in Right Side Frontoparietal Network in Primary Insomnia Patients.” European Radiology 28, no. 2: 664–672. 10.1007/s00330-017-5012-8. 28828546

[brb370088-bib-0027] Marques, D. R. , A. A. Gomes , G. Caetano , and M. Castelo‐Branco . 2018. “Insomnia Disorder and Brain's Default‐Mode Network.” Current Neurology and Neuroscience Reports 18, no. 8: 45. 10.1007/s11910-018-0861-3. 29886515

[brb370088-bib-0028] Menon, V. 2011. “Large‐Scale Brain Networks and Psychopathology: A Unifying Triple Network Model.” Trends in Cognitive Sciences 15, no. 10: 483–506. 10.1016/j.tics.2011.08.003. 21908230

[brb370088-bib-0029] Moreno‐Ortega, M. , A. Kangarlu , S. Lee , et al. 2020. “Parcel‐Guided rTMS for Depression.” Translational Psychiatry 10, no. 1: 283. 10.1038/s41398-020-00970-8. 32788580 PMC7423622

[brb370088-bib-0030] Nardone, R. , L. Sebastianelli , V. Versace , et al. 2020. “Effects of Repetitive Transcranial Magnetic Stimulation in Subjects With Sleep Disorders.” Sleep Medicine 71: 113–121. 10.1016/j.sleep.2020.01.028. 32173186

[brb370088-bib-0031] O'reardon, J. P. , H. B. Solvason , P. G. Janicak , et al. 2007. “Efficacy and Safety of Transcranial Magnetic Stimulation in the Acute Treatment of Major Depression: A Multisite Randomized Controlled Trial.” Biological Psychiatry 62, no. 11: 1208–1216. 10.1016/j.biopsych.2007.01.018. 17573044

[brb370088-bib-0032] Qi, S. , Y. Zhang , X. Li , et al. 2022. “Improved Functional Organization in Patients with Primary Insomnia after Individually‐Targeted Transcranial Magnetic Stimulation.” Frontiers in Neuroscience 16: 859440. 35360154 10.3389/fnins.2022.859440 PMC8960275

[brb370088-bib-0033] Riedel, M. , H.‐J. Möller , M. Obermeier , et al. 2010. “Response and Remission Criteria in Major Depression–A Validation of Current Practice.” Journal of Psychiatric Research 44, no. 15: 1063–1068. 10.1016/j.jpsychires.2010.03.006. 20447651

[brb370088-bib-0034] Rosen, A. C. , J. V. Bhat , V. A. Cardenas , et al. 2021. “Targeting Location Relates to Treatment Response in Active but Not Sham rTMS Stimulation.” Brain Stimulation 14, no. 3: 703–709. 10.1016/j.brs.2021.04.010. 33866020 PMC8884259

[brb370088-bib-0035] Rosenquist, P. B. , A. Krystal , K. L. Heart , M. A. Demitrack , and W. Vaughn Mccall . 2013. “Left Dorsolateral Prefrontal Transcranial Magnetic Stimulation (TMS): Sleep Factor Changes During Treatment in Patients With Pharmacoresistant Major Depressive Disorder.” Psychiatry Research 205, no. 1–2: 67–73. 10.1016/j.psychres.2012.09.011. 23021320

[brb370088-bib-0036] Rossi, S. , M. Hallett , P. M. Rossini , and A. Pascual‐Leone . 2009. “Safety, Ethical Considerations, and Application Guidelines for the Use of Transcranial Magnetic Stimulation in Clinical Practice and Research.” Clinical Neurophysiology 120, no. 12: 2008–2039. 10.1016/j.clinph.2009.08.016. 19833552 PMC3260536

[brb370088-bib-0037] Siddiqi, S. H. , S. F. Taylor , D. Cooke , A. Pascual‐Leone , M. S. George , and M. D. Fox . 2020. “Distinct Symptom‐Specific Treatment Targets for Circuit‐Based Neuromodulation.” American Journal of Psychiatry 177, no. 5: 435–446. 10.1176/appi.ajp.2019.19090915. 32160765 PMC8396109

[brb370088-bib-0038] Song, P. , H. Lin , S. Li , et al. 2019. “Repetitive Transcranial Magnetic Stimulation (rTMS) Modulates Time‐Varying Electroencephalography (EEG) Network in Primary Insomnia Patients: A TMS‐EEG Study.” Sleep Medicine 56: 157–163. 10.1016/j.sleep.2019.01.007. 30871961

[brb370088-bib-0039] Sonmez, A. I. , D. D. Camsari , A. L. Nandakumar , et al. 2019. “Accelerated TMS for Depression: A Systematic Review and Meta‐Analysis.” Psychiatry Research 273: 770–781. 10.1016/j.psychres.2018.12.041. 31207865 PMC6582998

[brb370088-bib-0040] Sonmez, A. I. , M. U. Kucuker , C. P. Lewis , et al. 2020. “Improvement in Hypersomnia With High Frequency Repetitive Transcranial Magnetic Stimulation in Depressed Adolescents: Preliminary Evidence From an Open‐Label Study.” Progress in Neuro‐Psychopharmacology & Biological Psychiatry 97: 109763. 10.1016/j.pnpbp.2019.109763. 31634515 PMC6904948

[brb370088-bib-0041] Tang, S. J. , J. Holle , O. Lesslar , C. Teo , M. Sughrue , and J. Yeung . 2022. “Improving Quality of Life Post‐Tumor Craniotomy Using Personalized, Parcel‐Guided TMS: Safety and Proof of Concept.” Journal of Neuro‐Oncology 160: 413–422. 10.1007/s11060-022-04160-y. 36308593

[brb370088-bib-0042] Tian, Y. , X. Chen , D. Xu , J. Yu , and X. Lei . 2020. “Connectivity Within the Default Mode Network Mediates the Association Between Chronotype and Sleep Quality.” Journal of Sleep Research 29, no. 5: e12948. 10.1111/jsr.12948. 31793113

[brb370088-bib-0043] Toussaint, A. , P. Hüsing , A. Gumz , et al. 2020. “Sensitivity to Change and Minimal Clinically Important Difference of the 7‐Item Generalized Anxiety Disorder Questionnaire (GAD‐7).” Journal of Affective Disorders 265: 395–401. 10.1016/j.jad.2020.01.032. 32090765

[brb370088-bib-0044] Voigt, J. D. , A. F. Leuchter , and L. L. Carpenter . 2021. “Theta Burst Stimulation for the Acute Treatment of Major Depressive Disorder: A Systematic Review and Meta‐Analysis.” Translational Psychiatry 11, no. 1: 330. 10.1038/s41398-021-01441-4. 34050123 PMC8163818

[brb370088-bib-0045] Wei, Y. , J. Leerssen , R. Wassing , D. Stoffers , J. Perrier , and E. J. W. Van Someren . 2020. “Reduced Dynamic Functional Connectivity Between Salience and Executive Brain Networks in Insomnia Disorder.” Journal of Sleep Research 29, no. 2: e12953. 10.1111/jsr.12953. 32164035 PMC7154624

[brb370088-bib-0046] Worley, S. L. 2018. “The Extraordinary Importance of Sleep: The Detrimental Effects of Inadequate Sleep on Health and Public Safety Drive an Explosion of Sleep Research.” Pharmacy and Therapeutics 43, no. 12: 758–763. 30559589 PMC6281147

[brb370088-bib-0047] Xiong, H. , R. J. Guo , and H. W. Shi . 2020. “Altered Default Mode Network and Salience Network Functional Connectivity in Patients With Generalized Anxiety Disorders: An ICA‐Based Resting‐State fMRI Study.” Evidence‐Based Complementary and Alternative Medicine 2020: 4048916. 10.1155/2020/4048916. 32855650 PMC7443230

[brb370088-bib-0048] Yesavage, J. A. , J. K. Fairchild , Z. Mi , et al. 2018. “Effect of Repetitive Transcranial Magnetic Stimulation on Treatment‐Resistant Major Depression in US Veterans: A Randomized Clinical Trial.” JAMA Psychiatry 75, no. 9: 884–893. 10.1001/jamapsychiatry.2018.1483. 29955803 PMC6142912

[brb370088-bib-0049] Yeung, J. T. , I. M. Young , S. Doyen , et al. 2021. “Changes in the Brain Connectome Following Repetitive Transcranial Magnetic Stimulation for Stroke Rehabilitation.” Cureus 13, no. 10: e19105. 10.7759/cureus.19105. 34858752 PMC8614179

[brb370088-bib-0050] Young, I. M. , H. M. Taylor , P. J. Nicholas , et al. 2023. “An Agile, Data‐Driven Approach for Target Selection in rTMS Therapy for Anxiety Symptoms: Proof of Concept and Preliminary Data for Two Novel Targets.” Brain and Behavior 13, no. 5: e2914. 10.1002/brb3.2914. 36949668 PMC10175990

[brb370088-bib-0051] Yu, S. , B. Guo , Z. Shen , et al. 2018. “The Imbalanced Anterior and Posterior Default Mode Network in the Primary Insomnia.” Journal of Psychiatric Research 103: 97–103. 10.1016/j.jpsychires.2018.05.013. 29804003

[brb370088-bib-0052] Zhou, F. , S. Huang , Y. Zhuang , L. Gao , and H. Gong . 2017. “Frequency‐Dependent Changes in Local Intrinsic Oscillations in Chronic Primary Insomnia: A Study of the Amplitude of Low‐Frequency Fluctuations in the Resting State.” NeuroImage: Clinical 15: 458–465. 10.1016/j.nicl.2016.05.011. 28649490 PMC5470569

[brb370088-bib-0053] Zhou, W.‐N. , H.‐Y. Fu , Y.‐F. Du , et al. 2016. “Short‐Term Effects of Repetitive Transcranial Magnetic Stimulation on Sleep Bruxism – A Pilot Study.” International Journal of Oral Science 8, no. 1: 61–65. 10.1038/ijos.2015.35. 27025267 PMC4822180

