# Supplementary material for: Improvements in Sleep Quality in Patients With Major Depressive and Generalized Anxiety Disorders Treated With Individualized, Parcel‐Guided Transcranial Magnetic Stimulation
Source: Brain Behav. 2024 Oct 17;14(10):e70088. doi: 10.1002/brb3.70088 (PMC11483549; doi:10.1002/brb3.70088)
Supplement: Supplementary file 1 — Figure S1 Clinical details and rTMS targets. Figure S2 Correlation of PSQI with EQ‐5D, BDI, and GAD‐7. [file BRB3-14-e70088-s001.docx]

| Patient Number | Age  (years) | Sex | Diagnosis | Duration of Symptoms (yrs) | Areas of Target |
| --- | --- | --- | --- | --- | --- |
| 1 | 48 | F | MDD | 10 | 1. L8Av  2. LPGs  3. RP47r* (iTBS 80%) |
| 2 | 33 | M | MDD | 11 | 1. L8Av 2. LPFm  3. L46 (Salience) |
| 3 | 22 | F | MDD with GAD | 3 | 1. L8Av  2. LPFm  3. LTE1m |
| 4 | 51 | M | MDD | 3 | 1. Ls6-8 2. LTe1m 3. RTe1m |
| 5 | 72 | F | MDD | 4 | 1. L8Av 2. LPFm 3. LTE1m |
| 6 | 73 | M | MDD | 30 | 1. L8Av  2. LPGs  3. RTE1m |
| 7 | 39 | F | MDD | 6 | 1. LPFm  2. LTE1m  3. RTe1m |
| 8 | 55 | M | MDD with GAD | 31 | 1. L8Av  2. LPGs  3. Rs6-8 |
| 9 | 54 | M | Moderate Depression with GAD | 39 | 1. L8Av 2. LPFm 3. LTE1m |
| 10 | 60 | M | MDD with GAD | 30 | 1. L8Av 2. LTE1m  3. RTE1m |
| 11 | 19 | F | OCD with GAD | 1 | 1. L6ma (Sensorimotor) 2. L8Av 3. LV4 (Visual Network) |
| 12 | 23 | M | PTSD with GAD | 3 | 1. L8Av 2. LPGs 3. LPFm |
| 13 | 49 | F | MDD with GAD | 22 | 1. L8Av 2. RTE1m 3. L43 |
| 14 | 62 | M | GAD | 0.25 | 1.Ls6-8 2. LPHT (Language Network) 3. LPFm |
| 15 | 41 | M | Moderate Depression | 2 | 1. L8Av 2. LPGs |
| 16 | 33 | F | GAD | 5 | 1. L8Av 2. RTE1m |
| 17 | 16 | F | MDD and ADHD | 9 | 1. L8Av  2. Ls6-8  3.LPGs |
| 18 | 46 | M | MDD and GAD | No information | 1. L8Av  2. Rs6-8  3.LPGs |
| 19 | 20 | M | MDD and GAD | 15 | 1. LPFM  2.LTE1m  3.RTe1m |
| 20 | 45 | M | MDD and GAD | 10 | 1.LTE1m  2. RTE1m  3. LPGs |
| 21 | 49 | M | MDD and GAD | 14 | 1. L8Av  2. RPFm  3.LPGs |
| 22 | 51 | M | MDD and GAD | 13 | 1. L8Av  2. RTE1m  3.LPGs |
| 23 | 33 | M | MDD | 8 | 1. Rs6-8  2.LPFm  3.LPGs |
| 24 | 39 | F | MDD | 5 | 1. L8Av  2. Ls6-8  3. LPGs |
| 25 | 52 | M | MDD | 32 | 1. LTE1m  2. Ls6-8  3. LPGs |
| 26 | 66 | M | MDD | 12 | 1. L8Av  2. Ls6-8  3. LPGs |
| 27 | 35 | F | MDD and GAD | 15 | 1. RTE1m  2. RPFm  3. Ls6-8 |

**Supplement 1. Clinical Details and rTMS Targets**

All other regions are part of CEN unless otherwise stated. All regions except patient 1’s RP47r (in asterisks) were targeted with cTBS at 80% of resting motor threshold.

**Supplement 2. Correlation of PSQI with EQ-5D, BDI, and GAD-7**

Correlation between PSQI and EQ-5D, BDI, and GAD-7 at baseline, post-treatment, and follow-up.

A) At baseline, PSQI was not correlated with EQ-5D (r = -0.2627, *p* = 0.1768).

B) At post-treatment, PSQI was not correlated with EQ-5D (r = -0.3351, *p* = 0.0875).

C) At follow-up, PSQI was significantly correlated with EQ-5D (r = -0.6702, *p* = 0.0012).

D) At baseline, PSQI was not correlated with BDI (r = 0.3458, *p* = 0.0904).

E) At post-treatment, PSQI was not correlated with BDI (r = 0.3450, *p* = 0.1069)

F) At follow-up, PSQI was significantly correlated with BDI (r = 0.6039, *p* = 0.0132).

G) At baseline, PSQI was not correlated with GAD-7 (r = 0.1601, *p* = 0.4880)

H) At post-treatment, PSQI was not correlated with GAD-7 (r = 0.4251, *p* = 0.0697).

H) At follow-up, PSQI was not correlated with GAD-7 (r = 0.5285, *p* = 0.0520).
